# Influence of Polycarboxylate Superplasticizers with Different Functional Units on the Early Hydration of C_3_A-Gypsum

**DOI:** 10.3390/ma12071132

**Published:** 2019-04-06

**Authors:** Kuangyi Hu, Zhenping Sun

**Affiliations:** 1 School of Materials Science and Engineering, Tongji University, Shanghai 200092, China; kadak.h@tongji.edu.cn; 2 Key Laboratory of Advanced Civil Engineering Materials of Ministry of Education, Tongji University, Shanghai 200092, China

**Keywords:** polycarboxylate superplasticizers, C_3_A-gypsum, functional units, hydration

## Abstract

Influence of polycarboxylate superplasticizers (PCs) with different functional units on the hydration heat and hydration products of C_3_A-gypsum was investigated. Three kinds of PCs with different monomers were discussed. It was seen that PCs mainly shortened the induction stage of C_3_A-gypsum hydration, and the amount of remaining gypsum was related to the duration of the induction stage. The second heat flow peak of the sample with PCs was higher than that of the blank. Moreover, PC intercalation occurred during the hydration.

## 1. Introduction

Tricalcium aluminate (3CaO∙Al_2_O_3_, C_3_A) is one of the essential components of Portland cement, which influences the early properties of cement pastes. Generally, calcium sulfate (CaSO_4_·H_2_O, gypsum) is added during the grinding period to adjust workability of the cement pastes before the final set. Therefore, the early hydration of cement depends on the C_3_A-gypsum reaction [1].

C_3_A reacts with water directly to produce calcium aluminate hydrates combined with different amounts of water, and all of these calcium aluminate hydrates ($C_{4}{\mathrm{AH}}_{13}$/$C_{4}{\mathrm{AH}}_{19}$ and $C_{2}{\mathrm{AH}}_{8}$, hydroxy-AFm) convert into hydrogarnet ($C_{3}{\mathrm{AH}}_{6}$, katoite) eventually. The equation for C_3_A hydration is shown below (Equation (1)):

(1) $$2C_{3}A+27H\to C_{4}{\mathrm{AH}}_{19}\left(C_{4}{\mathrm{AH}}_{13}\;\mathrm{when}\;R.H.\;<85\%\right)+C_{2}{\mathrm{AH}}_{8}\overset{R.T.}{\to }C_{3}{\mathrm{AH}}_{6}$$

where

R.H.—relative humidity,
R.T.—room temperature,
C—CaO,
A—Al_2_O_3_,
H—H_2_O.

C_3_A can also react with gypsum in water to form ettringite ($C_{6}{A\$}_{3}H_{32}$, AFt), which is presented as Equation (2):.

(2) $$C_{3}A+3{C\$H}_{2}+26H\to C_{6}{A\$}_{3}H_{32},$$

where:

$—SO_3_.

C_3_A turns AFt into AFm ($C_{4}{A\$H}_{12}$, kuzelite) when gypsum runs out, as in Equation (3):

(3) $$C_{3}A+C_{6}{A\$}_{3}H_{32}+4H\to 3C_{4}{A\$H}_{12},$$

Some researchers have studied the mechanisms and hydration process of C_3_A-gypsum systems [1,2,3,4,5,6,7]. Unlike in previous studies [2,3,4,5] showing that AFt or gel-like hydroxy-AFm could provide a substantial barrier to slow down the hydration rate of C_3_A in the presence of gypsum, the study of Minard et al. [6] conjectured that the early deceleration of C_3_A hydration might be caused by adsorption of sulfate ions on the surface dissolution sites of C_3_A particles. Based on the conclusions above, Quennoz and Scrivener [1] obtained new results showing that the rapid reaction after the exhaustion of gypsum was influenced by the surface area of C_3_A particles.

Nowadays, polycarboxylate superplasticizers (PCs) have been widely used in real concrete technology due to their favorable dispersibility. With their carboxylate backbone, PCs can be adsorbed on the surface of cement particles such that the steric hindrance provided by polyethylene oxide (EO) side chains can prevent the aggregation of particles during hydration. Hence, PCs influence not only the rheological behavior but also the hydration behavior of cement paste [8,9,10,11,12] due to its prolonged induction period of the early hydration of cement [8]. Thus, PCs may also have an impact on the C_3_A-gypsum early hydration.

The morphology and chemistry of C_3_A-gypsum-PCs hydrates were also studied [13,14,15]. Merlini et al. [13] found that superplasticizers had an effect on the total amount of formed ettringite in the C_3_A-gypsum system, and available sulfates in the system could be reduced via the formation of the organo-mineral phase. This kind of phase was determined with the dissolved sulfate concentration in the pore solution [14]. Moreover, Dalas et al. [15] demonstrated that the surface area of AFt was related to the electrostatic charge and dosage of PCs. While studies have been conducted on the adsorption behavior between superplasticizers and C_3_A clinkers [16,17], Alonso and Puertas [16] reported that the PCs molecular structure and the sulfate concentration in solution were the important factors that influence the adsorption behavior of PCs on the cubic C_3_A. Myers et al. [17] showed that the diffuse layer of C_3_A could weaken the dissolution-inhibition effects of superplasticizers.

Nonetheless, research has yet to reconcile the microscopic result and the macroscopic evidence of C_3_A-gypsum hydration with PCs, as well as those without. There is a lot that remains unknown about the effects of PCs, especially the effect of its different functional units on the C_3_A-gypsum hydration process. This paper proposes to gain further insight into the effects of PCs with different functional units on the hydration heat and hydration products of C_3_A-gypsum system, which could be helpful for understanding the working mechanism of PCs in the early hydration of cement.

## 2. Experimental 

### 2.1. Minerals (C_3_A/Gypsum)

C_3_A was provided by Dumaite (DMT, Shanghai, China), where X-ray diffraction (XRD) and Rietveld analyses (Topas Academic, Version 5) showed that the contained free-lime (CaO) was less than 1 wt%. The calcium sulfate dihydrate from Sinopharm Chemical Reagent (Shanghai, China) was also used in this study. Particle size distribution of minerals was analyzed with the LS 230 nanolaser particle size analyzer (Beckman Coulter, Brea, CA, USA), which is listed in Table 1.

### 2.2. PC Superplasticizers (PCs)

**Figure 1:**
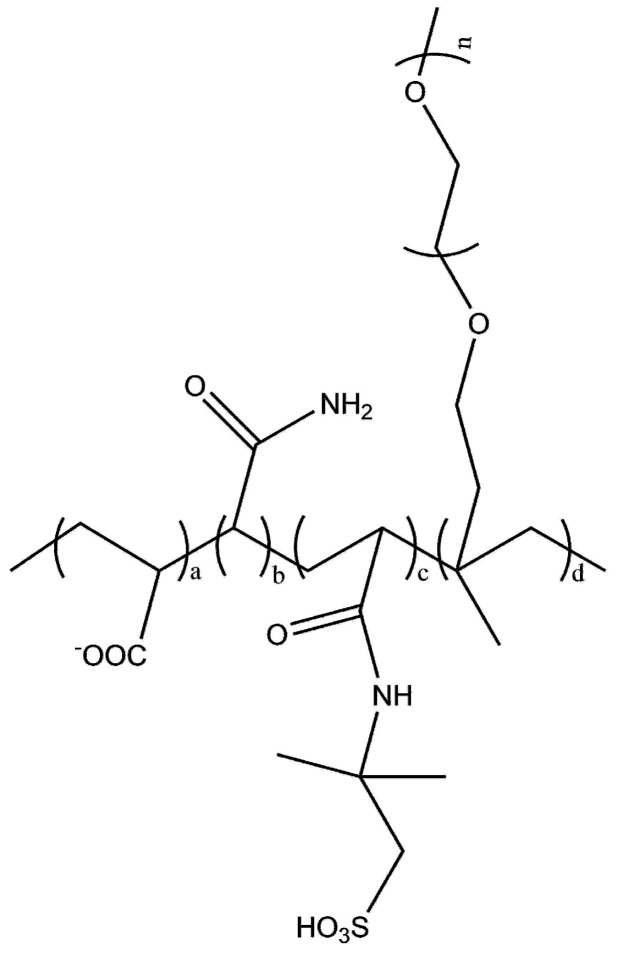
Molecular architecture of PCs.

Three PCs (PC1, PC2, and PC3) were synthesized via free-radical polymerization according to our previous work [18] and were employed in this study. The chemical structure of PCs is shown in Figure 1. VC (Vitamin C, Sinopharm Chemical Reagent, Shanghai, China)-H_2_O_2_ (Aladdin, Shanghai, China) were used as initiators, and TGA (Thioglycollic acid, Sinopharm Chemical Reagent, Shanghai, China) was used as the chain transfer agent to control the backbone length of PCs. Dosages of VC, H_2_O_2_, and TGA were 0.15 wt%, 1.00 wt%, and 0.35 wt% of the total weight of monomers, respectively. TPEG2400 (Isoprenyl oxy poly(ethylene glycol) ether with molecular weight of 2400, Levima, Tengzhou, China) as macromonomer was diluted into 40 wt% water solution in a 250 mL four-neck round-bottom flask equipped with a constant stirrer and was heated in the 45 °C water bath. AA (Acrylic acid, Sinopharm Chemical Reagent, Shanghai, China) as a carboxylate monomer was diluted into 40 wt% solution in beaker A. AM (Acrylamide, Aladdin, Shanghai, China) or AMPS (2-acrylamido-2-methylpropanesulfonic acid, Aladdin, Shanghai, China) as a functional monomer was diluted into 40 wt% solution in beaker B. VC and TGA were mixed and diluted into a 40 wt% solution in beaker C. H_2_O_2_ was added into TPEG2400 solution all at once when TPEG solution was heated up to 45 °C. Then solution in beakers A, B, and C were fed dropwise with peristaltic pumps into TPEG solution for 3.0 h, 3.0 h, and 3.5 h respectively. The mixture in flask was stirred at 45 °C for an extra hour in order to react completely. Gel permeation chromatography (GPC) analysis of the synthesized PCs was performed using Waters Alliance 2695 instrument (Waters, Eschborn, Germany). Details of the monomer ratios and the molecular characteristics are shown in Table 2.

### 2.3. Preparation of C_3_A-Gypsum Samples

Samples of C_3_A in the absence and presence of various amount of calcium sulfate were prepared for study. Mix ratios were presented in Table 3. The powders were dry-mixed manually for 10 min in a mortar before mixed with deionized water or PC solutions.

### 2.4. Hydration Heat

The hydration heat of C_3_A-gypsum pastes was measured using the TAM Air isothermal calorimeter (TA instrument, New Castle, PA, USA) at 20 °C. Pastes were prepared outside the calorimeter and were introduced in the calorimeter immediately after mixing (less than 2 min).

### 2.5. XRD Analysis

The traces of sulfur component in PC3 samples might contaminate the platinum modules during heating process in TGA analysis. Furthermore, accurate crystallographic information of some C_3_A-gypsum hydrates was unknown for QXRD (Quantitative X-ray Diffraction)/Rietveld analysis. Thus, it was not possible to calculate absolute amounts of phases in this study. Semi-quantitative method could be an alternative option to reveal the trend changes of phases.

Samples listed in Table 3 were mixed separately in sealed containers and cured at (20 ± 1) °C. Ethyl alcohol was added into containers immediately after 24 h of hydration to stop the reaction. Then samples were dried at 45 °C for 48 h in the vacuum drying oven (Shengke, Shanghai, China) before the test. XRD patterns were obtained using the Bruker AXS D8 (Karlsruhe, Germany) with a RINT2000 vertical goniometer operating at 40 kV and 250 mA. A CuKα source with a 0.3 mm slit was used. The scan was performed at the 2θ angles between 5° and 40° with an increment of 0.02°.

As for peak area (in counts) of phases, the specific diffraction peak was fitted with a Gaussian function to make it smoother first. Two end points of the smoothed curve were used as the baseline. Then, the peak area between the smoothed curve and baseline was calculated by integrating over the specific 2θ angles range. For ettringite, the preferred 2θ angles were between 8.8° and 9.4°. The preferred 2θ angles of katoite were between 39.0° and 39.6°. For kuzelite, the preferred 2θ angles were between 9.6° and 10.2°. Finally, for gypsum, the preferred 2θ angles were between 11.4° and 12.0°.

## 3. Results and Discussion

### 3.1. Early Hydration of C_3_A-Gypsum System

**Figure 2:**
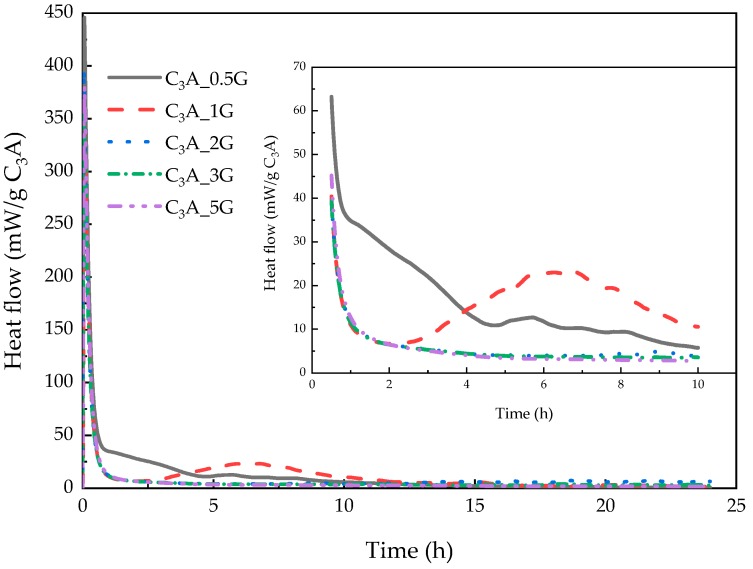
Heat evolution curves of C_3_A with various amounts of gypsum during the 24-h hydration.

To begin with, the hydration of C_3_A in the presence of various amount of gypsum was investigated. The hydration heat evolution curves of C_3_A with the gypsum/C_3_A molar ratios (G/C) between 0.5 and 5 during 24 h are plotted on Figure 2. Two exothermic peaks were observed when the G/C ratio was less than 2. It was shown that the first one was extremely sharp and the second one was relatively broad. However, there was only one exothermic peak during the 24 h when more gypsum was added. Therefore, in accordance with previous research [1,6], this phenomenon is due to the higher gypsum dosage and liquid to solid (L/S) ratio.

**Figure 3:**
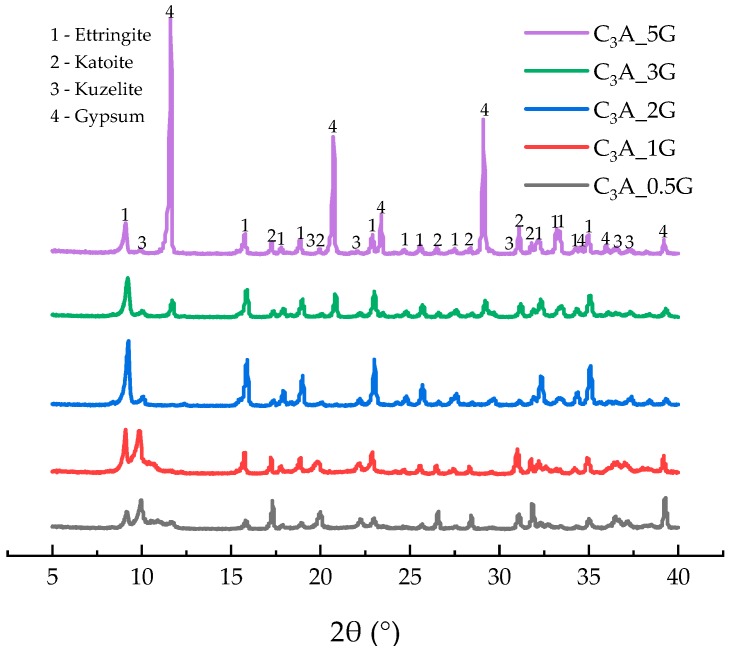
XRD patterns of phases obtained from C_3_A with various amounts of gypsum after the 24-h hydration.

Figure 3 showed the XRD results of the hydrates after the 24-h hydration of C_3_A-gypsum. Excessive gypsum was found when the G/C ratio was higher than 2. AFm was detected in all samples even when there was excessive gypsum in the system. However, this result is contradictory to what we have known from Reference [2] showing that AFm forms only when gypsum is exhausted, as we summarized in the Introduction section.

Based on the shape of the hydration heat curves, the hydration age can be divided into three stages, which are the dissolution–crystallization stage, the induction stage, and the transformation stage with the exhaustion of gypsum, respectively.

#### 3.1.1. Dissolution–Crystallization Stage

The dissolution–crystallization stage includes the first exothermic peak. After C_3_A-gypsum is in contact with water, hydroxy-AFm and AFt are both precipitated due to the different dissolution rates of C_3_A and gypsum. As can be seen from Figure 2, the first peak width of each curve is nearly the same, which means the dosage of gypsum had no impact on the duration of this stage. Different heights of the first peaks could be caused by the measuring error of the C_3_A weight, the different interval between sample mixing time and calorimeter measuring time, and the various reaction heats of Equations (1) and (2).

Because of the low solubility of gypsum, hydroxy-AFm was first precipitated by the dissolution of C_3_A. Furthermore, the formation of ettringite was the reason why consumption rate of sulfate ion was higher than the dissolution rate of gypsum [6]. Therefore, the dissolution of C_3_A could be the main factor that controlled the reaction rate during this stage. The specific surface area of C_3_A could have determined the hydration rate of C_3_A-gypsum system [1,6]. Some researchers believed that sulfate ions could combine with hydroxy-AFm rather than C_3_A, which could explain the time interval between the dissolution of gypsum and the formation of ettringite.

#### 3.1.2. Induction Stage

When gypsum is not overdosed, unhydrated C_3_A would consume AFt, and as a result, AFm would be precipitated, and two thermal peaks would be found in the hydration heat flow curves as well. The interval between the two peaks is commonly known as the induction stage when the hydration rate is temporarily low and would be accelerated again in the following hours. As illustrated in Figure 2, only sample C_3_A_1G had a complete induction period during the 24-h hydration.

The formed hydrates could cover the whole surface of C_3_A particles, thus they inhibit the hydration of C_3_A, which was the primary explanation for the induction stage in past decades [2,3,4,5]. However, the morphology of ettringite was considered unlikely to block the ion exchange [5]. Minard et al. [6] found that the hydroxy-AFm “sheet” could be present on the surface of C_3_A after a 3-min hydration of C_3_A-gypsum. However, platelets of the same precipitation was not observed after 5-min hydration of C_3_A-hemihydrate [7]. Therefore, the hydrates after dissolution–crystallization stage may not be the critical reason for a slowdown of the hydration of C_3_A-gypsum. Afterwards, the new kinetics was put forward and adsorption of SO_4_^2−^ onto etch pit sites of C_3_A particles was thought to be the main reason for the control of the reaction during the induction period [1,6]. Considering this result, an important characteristic at the end of the induction stage was the complete disappearance of sulfate ion [6]. It is clear that the formation of AFt continuously occurred with a slow rate during the whole stage. Nevertheless, when the G/C ratio increased from 0.5 to 5, the induction stage only existed in a range where gypsum was insufficient, as shown in Figure 2, which means the adsorption of sulfate ions caused a dynamic equilibrium between the dissolution of C_3_A and the consumption of sulfate ions in solution when gypsum was ample, AFt formed with an almost constant rate until C_3_A ran out, and no other visible exothermic peaks could be observed except the first one. The exhaustion of sulfate ions in the media could induce the desorption of species in order to maintain the equilibrium mentioned above. Therefore, the dissolution of C_3_A increased again and a second hydration exothermic peak was observed. The amount of sulfate ions hence determined the duration of this stage. Adsorbed sulfate ions on the active site of C_3_A could reduce the dissolution rate of C_3_A as well.

#### 3.1.3. Transformation Stage

After the gypsum depletion, C_3_A could be consumed by AFt and the reaction in Equation (3) would proceed during the transformation stage, which included the second hydration exothermic peak. As depicted in Figure 2, with the increases of sulfate ions, the second exothermic peak of samples was delayed and reduced when gypsum was exhausted.

Regarding this stage, the work of Quennoz et al. [1] reported that the remaining site for AFm nucleation on the surface of C_3_A controlled the acceleration part, and the space available for growth was the main factor impacting the slope of the deceleration part. Also, the result of Kirchheim et al. [19] proved that only the reaction Equation (3) existed during this stage instead of the concurrence of Equations (1) and (3).

### 3.2. Hydration of C_3_A-Gypsum in the Presence of PCs

Two kinds of functional units are discussed in this study. PC2 contains AM with an amide unit as the first functional unit. PC3 contains AMPS with the same molar ratio of the amide unit and sulfo unit. The sulfo unit is the second functional unit. PC1 was synthesized with only carboxylate monomers.

#### 3.2.1. Effects of PC with Only Carboxylate Monomers

**Figure 4:**
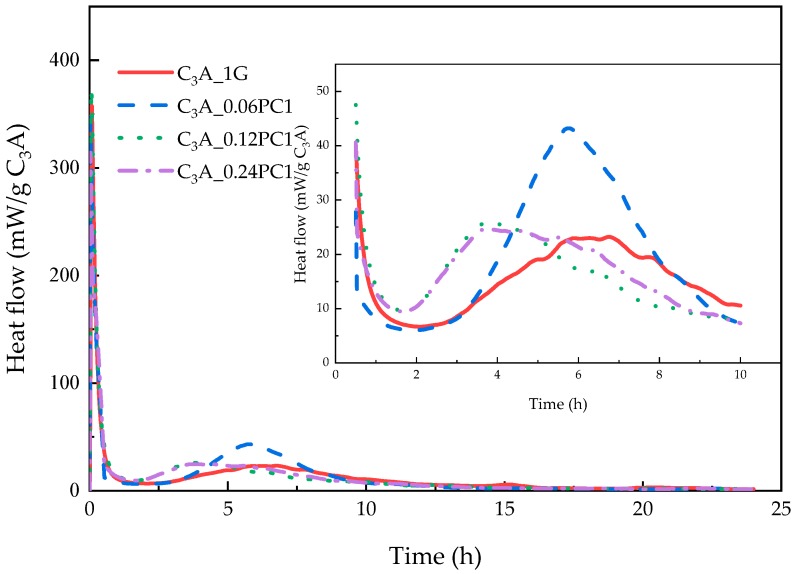
Heat evolution curves of C_3_A-gypsum with different concentrations of PC1 solution during the 24-h hydration.

**Figure 5:**
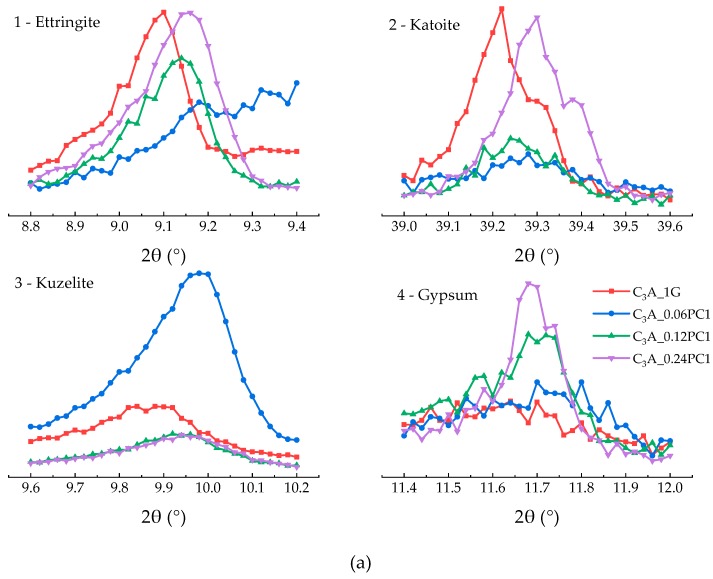

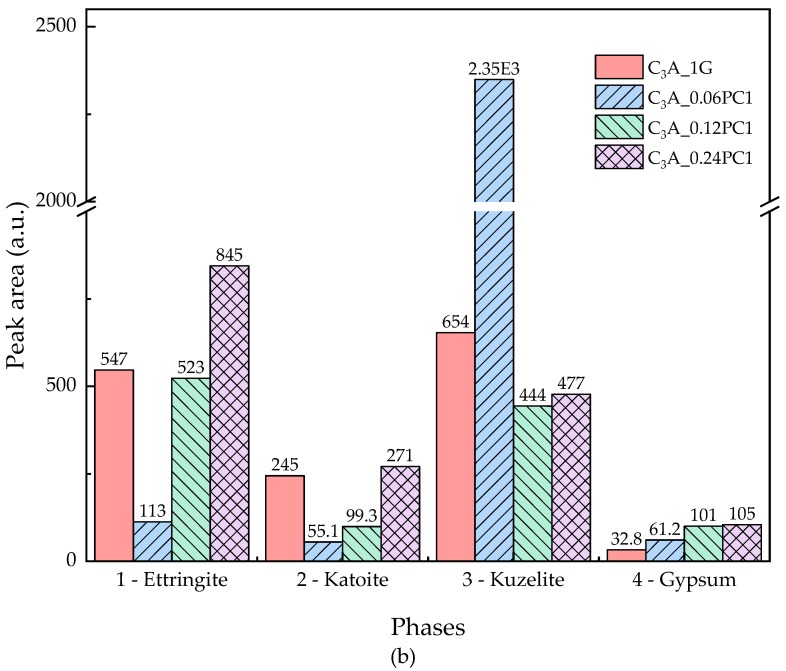
(**a**) XRD patterns of phases obtained from C_3_A-gypsum with different concentrations of PC1 solution after the 24-h hydration. (**b**) XRD peak area of each phase in (a).

The hydration heat of C_3_A-gypsum mixed with different concentration of PC1 solutions within 24 h is demonstrated in Figure 4. As depicted in Figure 4, low concentration of PC1 could enhance the transformation of AFt not only by cutting the induction stage but by increasing the second heat flow peak compared to the sample without PCs. With the increased concentration of PC1, the second heat flow peak first increased and then decreased, and the induction stage was shortened as well. Samples with 0.12% and 0.24% concentration of PC1 had a subtle difference on the second heat flow peak, but the slopes of the deceleration part were not the same. The XRD patterns of phases after the 24-h hydration are indicated in Figure 5a. It was noticed that the 2θ value of hydrates formed via samples mixed with PC1 solution had little shift compared with the same hydrates produced using the blank sample. Carboxylate units could be adsorbed on the surface of positive charge particles, which included C_3_A and hydrates such as hydroxy-AFm [10,14,16]. Therefore, the peak shift may reveal that the PC intercalation [13,14] occurred during C_3_A-gypsum hydration. The area of phase peaks in Figure 5a is shown in Figure 5b. With the increased dosage of PC1, more ettringite remained with a lower gypsum cost. Samples with 0.06% concentration of PC1 had the highest production of AFm, but the product of hydroxy-AFm was the least.

1. Dissolution–crystallization stage

PC1 molecules could be adsorbed onto the surface of C_3_A particles after the mixing step. The more PC1 molecules that were introduced, the more they were adsorbed onto the C_3_A particles before reaching the saturated adsorption. With the increased concentration of PC1, more and more PC1 molecules occupied the dissolution sites on the surface of C_3_A, and the size effect of adsorbed PC1 molecules may have reduced the nucleation and growth of hydrates such that the precipitation of hydroxy-AFm at the beginning of hydration was hindered. On the other hand, EO chains could attract more water molecules to enhance the ion exchange between solution and solids [8], which was due to the characteristics of the surfactant. Both factors are the reasonable cause of the similar production of hydroxy-AFm in the blank sample and sample with a 0.24% concentration of PC1.

2. Induction stage

The dosage of PC1 may have effected the induction stage, which was reflected in the duration between the two exothermic peaks. More PC1 advanced the transformation stage, probably by inhibiting the dissolution of gypsum, which could be concluded from the remains of gypsum, as shown in Figure 5b. The concentration of unconstrained PC1 molecules determine the release of sulfate ions with negative charge. Despite the fact that free PC1 might have expended Ca^2+^ in solution [20], the original equilibrium between the formation of AFt and the dissolution of gypsum may have been broken by PC1 and the new dynamic equilibrium about the dissolution of C_3_A, the release of sulfate ions, and the consumption of free PC1 molecules was possibly established. With the exhaustion of PC1, the dissolution of C_3_A increased again and AFm was formed because the activation energy of Equation (1) was higher than that of Equation (2) [1] such that gypsum remained.

3. Transformation stage

PCs had an influence on the morphology of AFt via hindering the growth of crystals and increasing the surface area [15]. After depletion of the dissociative sulfate ions, the interaction between the small ettringite particles with substantial specific surface area and the unhydrated C_3_A was enhanced under the influence of adsorbed PC molecules on both surfaces. This was probably the reason why the heat flow peak was higher and narrower than the blank sample. Samples with less PC1 dosage may have produced more AFt before the transformation stage, while the dissolution of gypsum was restrained. Hence, the highest AFm production, from the sample with 0.06% concentration of PC1, could be possible.

#### 3.2.2. Effects of PC with AM Monomers

**Figure 6:**
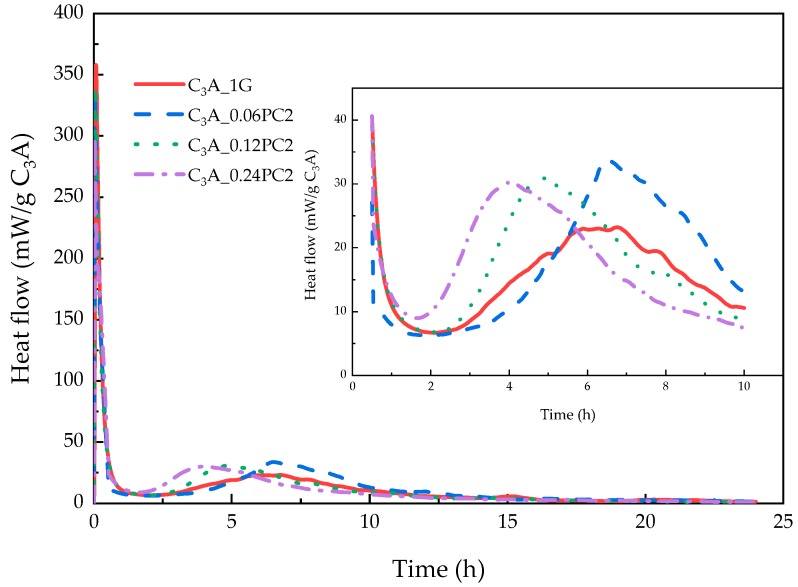
Heat evolution curves of C_3_A-gypsum with different concentrations of PC2 solution during the 24-h hydration.

**Figure 7:**
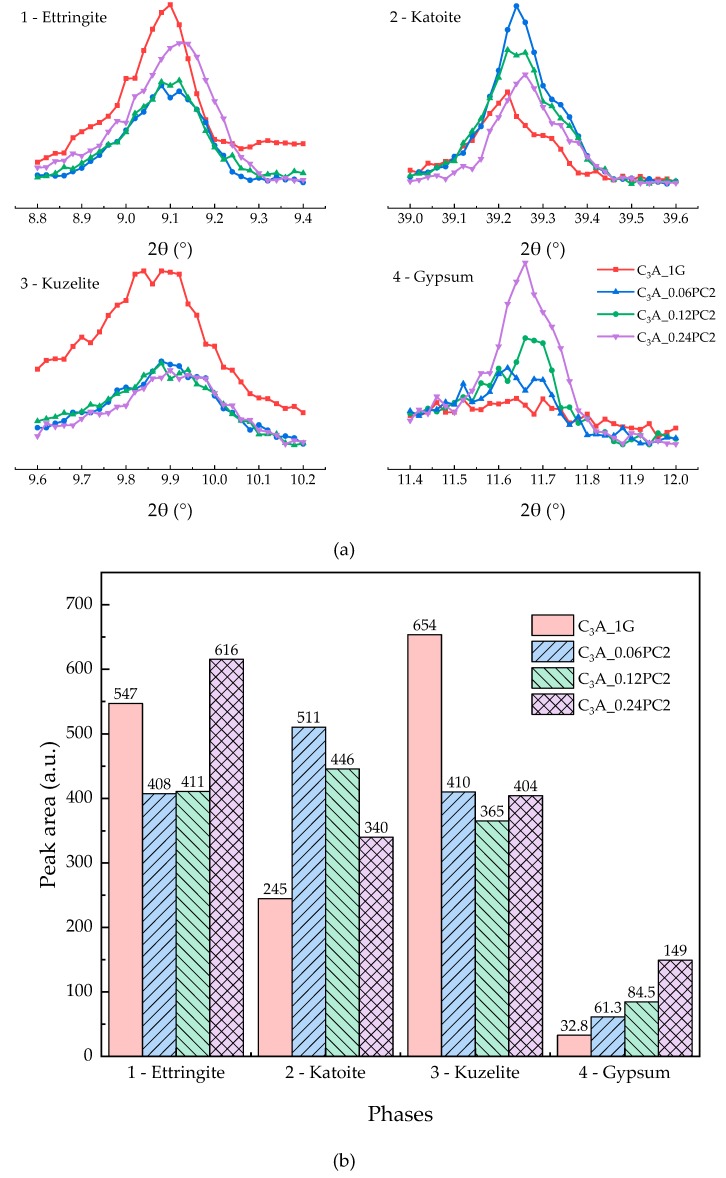
(**a**) XRD patterns of phases obtained from C_3_A-gypsum with different concentrations of PC2 solution after the 24-h hydration. (**b**) XRD peak area of each phase in (a).

The hydration heat of C_3_A-gypsum mixed with a different concentration of PC2 solutions within 24 h is demonstrated in Figure 6. It is apparent from Figure 6 that with the increased PC2 dosage, the major differences were concentrated upon the induction stage and the transformation stage. A 0.06% concentration of PC2 could prolong the induction stage, but more PC2 might shorten that. No matter how many PC2 molecules were introduced in the hydration of C_3_A-gypsum, exothermic peaks in the transformation stage had a minor difference, yet higher than the blank sample. XRD patterns of C_3_A-gypsum hydration with different PC2 concentration are revealed in Figure 7a. Furthermore, the area of phase peaks in Figure 7a is shown in Figure 7b. The amount of ettringite was decreased first and then increased in the peak area with the increased PC2 dosage. The amount of hydroxy-AFm created via samples with PC2 was more than that of the blank sample. With the increased PC2 concentration, the production of AFm was nearly the same, which was still less than that of the blank sample.

Based on articles published in public [21], the amide unit is nonionic unit that can hardly be adsorbed on the surface of particles. Therefore, the adsorption amount of PC2 on the surface of C_3_A particles was less than that of PC1 under the same PC concentration. This was owed to the lower electrostatic charge of the PC2 backbone. Fewer adsorbed PC2 molecules on the clinker surface may have released more dissolution sites such that the precipitation of hydroxy-AFm was promoted. Furthermore, the hydrogen bond between the water molecule and amide unit also enhanced the hydration of C_3_A. On the other hand, the residual gypsum increased as a result of more unconstrained PC2 molecules in the media. That means that the induction stage was shortened with increased PC2 concentration. The AFm production of samples with PC2 was less than that of the blank sample, where this may be attributed to the lack of C_3_A. PC2 with a lower electrostatic charge than PC1 could have a weaker inhibition effect on the dissolution of gypsum such that the AFt formation rate of samples with PC2 was higher than that of samples of PC1. The precipitation of hydroxy-AFm and AFt consumed more C_3_A with PC2 addition during the dissolution–crystallization stage and the induction stage, and as a consequence, the formation of AFm in the transformation stage was less than the blank.

#### 3.2.3. Effects of PC with AMPS Monomers

**Figure 8:**
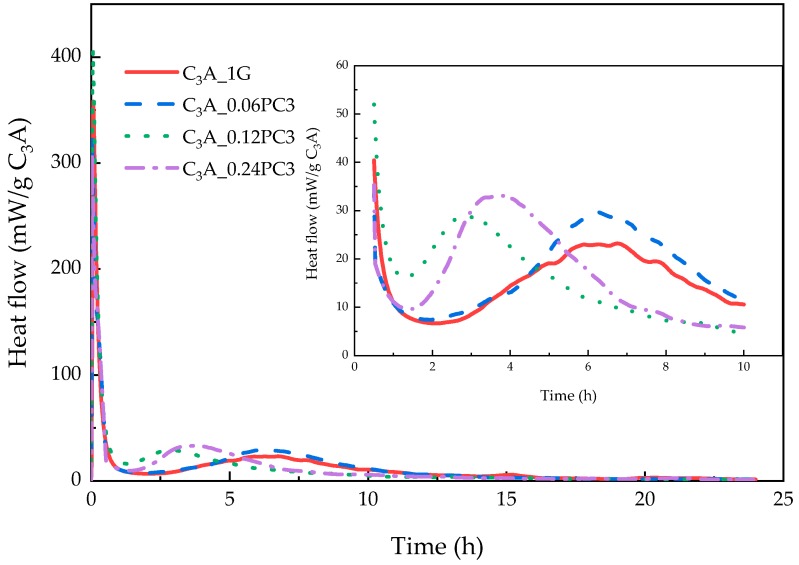
Heat evolution curves of C_3_A-gypsum with different concentrations of PC3 solution during the 24-h hydration.

**Figure 9:**
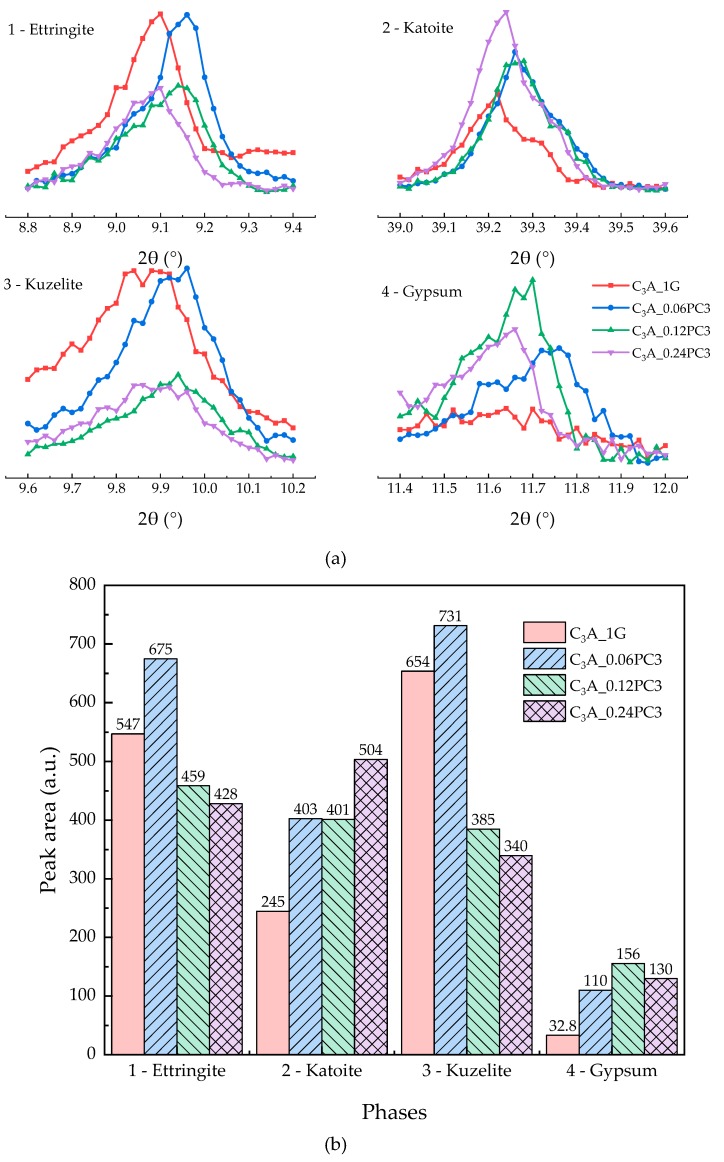
(**a**) XRD patterns of phases obtained from C_3_A-gypsum with different concentrations of PC3 solution after the 24-h hydration. (**b**) XRD peak area of each phase in (a).

The hydration heat of C_3_A-gypsum mixed with different concentration of PC3 solutions within 24 h is demonstrated in Figure 8. As can be seen from Figure 8, although AMPS contained the amide unit, the hydration heat evolution of C_3_A-gypsum with PC3 varied from that with PC2. With the increased PC3 concentration, the second exothermic peak of each sample rose, but the order of appearance time of the transformation heat flow peak was different. XRD patterns of phases after the 24-h hydration are shown in Figure 9a. The peak area of phases in Figure 9a is shown in Figure 9b. The sample with a 0.12% concentration of PC3 had the shortest induction stage, with the most gypsum remaining. Although samples with 0.12% and 0.24% concentration of PC3 released more heat during the transformation stage, the produced AFm and remaining AFt were less than the blank sample and the sample with a 0.06% concentration of PC3.

Unlike the amide unit, the sulfo unit is easier to ionize; furthermore, its hydrophilicity is better than the carboxylate unit [8]. Although the adsorbed PC3 molecules occupy lots of active sites on the surface of C_3_A, like PC1, their excellent hydrophilicity resulted in an attraction for more water molecules such that the ion transport between the clinker and solution was accelerated. Therefore, a low concentration of PC3 may enhance the production of hydroxy-AFm without reducing the formation of AFt and AFm. When the concentration of PC3 increased, more unconstrained PC3 molecules hindered the dissolution of gypsum, the induction stage was shortened, and the production of AFt and AFm decreased as well.

As the concentration of PC3 continually rose, the sample with a 0.24% concentration of PC3 had a longer induction stage than the sample with a 0.12% concentration of PC3. Furthermore, the residual gypsum after the 24-h hydration decreased. This phenomenon might be explained with a proper hypothesis about the critical micelle concentration (CMC) of PC3. Active sites of created hydrates were mostly covered with PC3 molecules such that the induction stage would be prolonged until PC3 molecules were depleted via intercalation and complexation with Ca^2+^.

#### 3.2.4. Effect of the PCs with Different Functional Units

**Figure 10:**
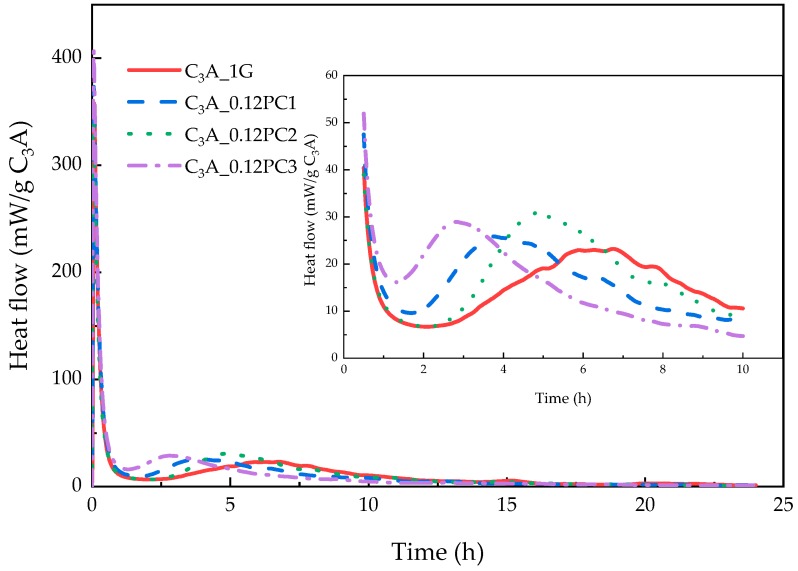
Heat evolution curves of C_3_A-gypsum with a 0.12% concentration of different kinds of PCs solution during the 24-h hydration.

**Figure 11:**
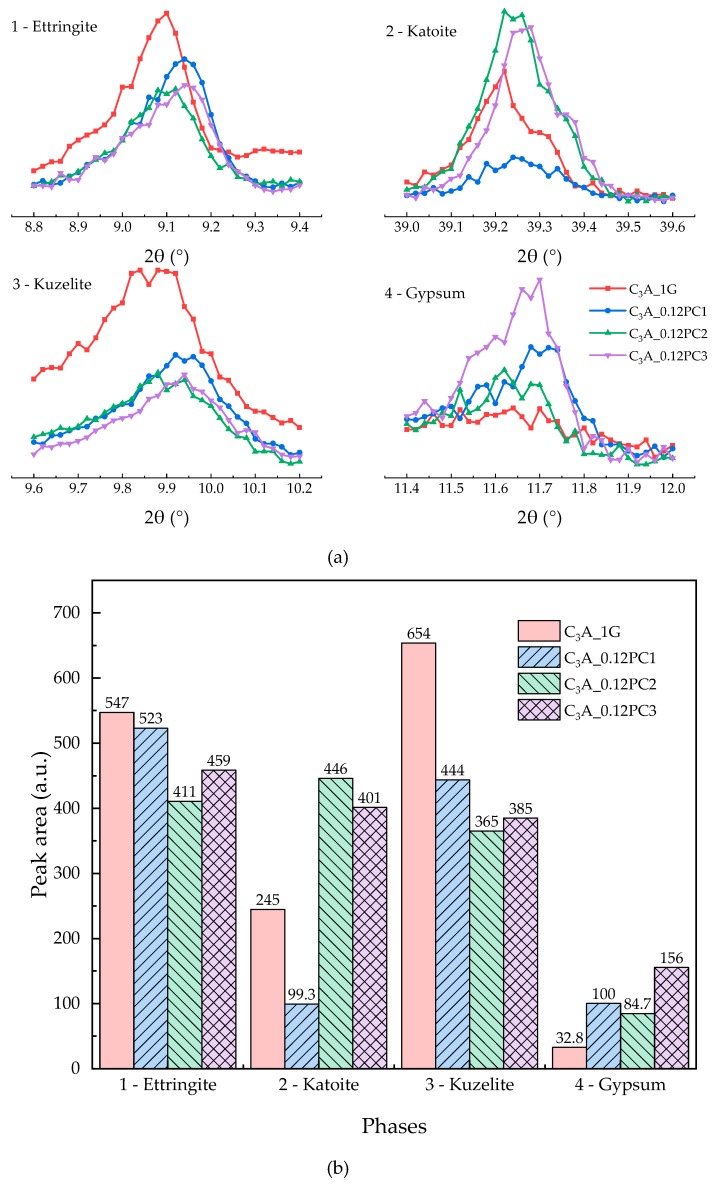
(**a**) XRD patterns of phases obtained from C_3_A-gypsum with the same concentrations of different kinds of PCs solution after the 24-h hydration. (**b**) XRD peak area of each phase in (a).

The hydration heat of C_3_A-gypsum mixed with a 0.12% concentration of PCs with different functional units within 24 h is revealed in Figure 10. According to Figure 10, PCs shortened the induction stage of C_3_A-gypsum hydration. PC copolymerized with AMPS could tremendously cut down on the induction stage. The second heat flow peaks of the samples with PCs was higher than that of the blank sample. XRD patterns of phases after the 24-h hydration are illustrated in Figure 11a. All the hydrates formed by samples with PCs displayed a shift of the intensity peaks, which indicated that PC intercalation occurred during C_3_A-gypsum hydration. Different shifts caused by different kinds of PCs probably meant the conformation of intercalated PC molecules was different. This agreed well with results reported by Plank et al. [9]. The area of the XRD phase peaks in Figure 11a is shown in Figure 11b. Except for PC1, PCs clearly promoted the formation of hydrogarnet. The dissolution of gypsum was inhibited by PCs such that the formation of AFt was restrained. Gypsum remained in the system after the 24-h hydration, which was consistent with the duration of the induction stage of samples.

Although all the functional units mentioned may reduce the adsorption amount of PCs on the surface of clinkers, different functional units had varying influences on the hydration of C_3_A-gypsum. PC1 molecules could be primarily adsorbed onto the surface of clinkers because of electrostatic interaction when C_3_A-gypsum mixed with the PCs–water solution [22]. Active sites on the surface of C_3_A were occupied such that the precipitation of hydroxy-AFm of the sample with PC1 was slowed down. Other PC molecules might have mainly been still in solution due to the lack of carboxylate units. The formation of hydroxy-AFm was advanced with other PCs because the adsorbed PC molecules could attract more water molecules to accelerate the dissolution of C_3_A [8]. PC molecules with negative electrostatic charge in solution prevented gypsum from dissolving and occupying dissolution sites on the surface of clinkers that were originally for sulfate ions. Therefore, the formation of AFt during the induction stage is hindered, the duration of the induction stage was prolonged as well. PC molecules with different amounts of electrostatic charge had different abilities to inhibit sulfate ions from releasing. PC3 with sulfo units had the most amount of negative electrostatic charge among them. Hence, the sample with PC3 had the shortest induction stage during C_3_A-gypsum hydration. The produced AFt particles were small but had higher specific surface area [15], which was owed to the steric hindrance and repulsive interaction [23,24] of adsorbed PCs. Furthermore, this is a possible reason for the higher exothermic peak during the transformation stage than the blank sample.

## 4. Conclusions

First, hydration of C_3_A in the presence of various amounts of gypsum was studied. Based on the hydration heat evolution curves, three stages were proposed in an attempt to elaborate the early hydration behavior of C_3_A-gypsum:

- In the dissolution–crystallization stage, the precipitation of hydroxy-AFm and AFt coexisted, although there may be a time interval between them. The dosage of gypsum could not have a significant impact on the duration of this stage.
- Unhydrated C_3_A remained after the sulfate depletion was the precondition of the induction stage, and the specific G/C ratio allowing the induction stage could exist was hard to obtain because of the uncertain consumption of C_3_A or gypsum during the dissolution–crystallization. The duration of this stage depended on the number of soluble sulfate ions.
- The transformation stage started after the sulfate exhaustion when AFt was converted into AFm. Remaining C_3_A after the induction stage determined the duration of this stage.

Influences of PCs with different functional units on hydration behavior of C_3_A-gypsum was investigated using isothermal calorimetry and XRD analysis:

- A low concentration of PC, which copolymerized only with carboxylate unit in solution, could promote the transformation stage of C_3_A-gypsum hydration significantly. With the increased concentration of PC1, the appearance of second heat flow peak advanced, the amount of remained gypsum increased, and the amount of remained AFt increased.
- The concentration of PC copolymerized with an amide unit influenced the duration of the induction stage. A low concentration of PC2 could prolong it. PC2 may have accelerated the formation of hydrogarnet. Although the amount of AFm created was less than the blank sample, a higher transformation heat flow peak could be found during the hydration of samples with PC2.
- As the concentration of PC that copolymerized with AMPS monomers increased, the duration of the induction stage was shortened first, and then prolonged. A low concentration of PC3 had a minor influence on the hydration of C_3_A-gypsum, except for the exotherm during the transformation stage. Also, the dosage of PC3 could enhance the hydration of C_3_A such that the production of hydrogarnet was more than the blank sample.

In general, the induction stage of C_3_A-gypsum hydration may have been shortened with the PCs addition, and the amount of remaining gypsum was related to the duration of the induction stage. Moreover, the exothermic peak in the transformation stage was higher than the blank sample. PC intercalation existed during the hydration.

## Figures and Tables

**Table 1:** Particle size distribution of minerals.

Minerals	Diameter (μm)			
	d_10_	d_50_	d_90_	d_m_
C_3_A	3.09	6.88	15.92	8.83
Gypsum	8.10	20.16	38.00	21.79

Note: d_10_, d_50_, and d_90_ represent the particle size below which the volume percent is 10%, 50%, and 90%, respectively; d_m_ is the mean particle size.

**Table 2:** Monomers molar ratio and molecular data of PCs.

Sample	The Molar Ratio of Monomers				Molecular Characteristics		
	AA	AM	AMPS	TPEG2400	M_n_ (Da)	M_w_ (Da)	PDI
PC1	5	0	0	1	48,600	107,400	2.21
PC2	1	4	0	1	35,740	67,840	1.90
PC3	1	0	4	1	36,030	83,110	2.31

Note: M_n_ means number average molecular mass, M_w_ means weight average molecular mass, and PDI means polydisperse index.

**Table 3:** Summary of the C_3_A-gypsum samples.

Samples	C_3_A (g)	Gypsum (g)	Deionized Water/PC Solutions
C_3_A_0.5G	1 (3.7 mmol)	0.385 (1.9 mmol)	10 g deionized water
C_3_A_1G	1 (3.7 mmol)	0.770 (3.7 mmol)	10 g deionized water
C_3_A_2G	1 (3.7 mmol)	1.540 (7.4 mmol)	10 g deionized water
C_3_A_3G	1 (3.7 mmol)	2.310 (11.1 mmol)	10 g deionized water
C_3_A_5G	1 (3.7 mmol)	2.310 (18.5 mmol)	10 g deionized water
C_3_A_0.06PC1	1 (3.7 mmol)	0.770 (3.7 mmol)	10 g 0.06% PC1 solution
C_3_A_0.12PC1	1 (3.7 mmol)	0.770 (3.7 mmol)	10 g 0.12% PC1 solution
C_3_A_0.24PC1	1 (3.7 mmol)	0.770 (3.7 mmol)	10 g 0.24% PC1 solution
C_3_A_0.06PC2	1 (3.7 mmol)	0.770 (3.7 mmol)	10 g 0.06% PC2 solution
C_3_A_0.12PC2	1 (3.7 mmol)	0.770 (3.7 mmol)	10 g 0.12% PC2 solution
C_3_A_0.24PC2	1 (3.7 mmol)	0.770 (3.7 mmol)	10 g 0.24% PC2 solution
C_3_A_0.06PC3	1 (3.7 mmol)	0.770 (3.7 mmol)	10 g 0.06% PC3 solution
C_3_A_0.12PC3	1 (3.7 mmol)	0.770 (3.7 mmol)	10 g 0.12% PC3 solution
C_3_A_0.24PC3	1 (3.7 mmol)	0.770 (3.7 mmol)	10 g 0.24% PC3 solution
